# Unintentional Injuries and Sociodemographic Factors among Households in Ethiopia

**DOI:** 10.1155/2020/1587654

**Published:** 2020-12-03

**Authors:** Hailemichael Mulugeta, Yifokire Tefera, Teferi Abegaz, Steven M. Thygerson

**Affiliations:** ^1^College of Health Science, Debre Berhan University, Debre Berhan, Ethiopia; ^2^School of Public Health, College of Health Sciences Addis Ababa University, Addis Ababa, Ethiopia; ^3^Department of Public Health, College of Life Sciences, Brigham Young University, Provo, UT, USA

## Abstract

**Background:**

Unintentional injuries are a public health problem throughout the world including Africa. Most of the injury studies in Ethiopia are from the healthcare facility or workplace that does not reflect the problem at the community level. Therefore, this study aimed to assess the unintentional injuries and sociodemographic factors among households in Ethiopia.

**Methods:**

This study was done from the Ethiopian Demographic and Health Survey conducted in 2016. The survey collected information about unintentional injuries and injury mechanisms in the past 12 months among 16,650 households. The selection of households was from nine regions and two city administrations of Ethiopia using a stratified cluster sampling procedure. Descriptive statistics were used to characterize the data and the chi-square test was applied as a test of significance and a *p* value of <0.05 was considered statistically significant.

**Result:**

Of the 16,650 households that participated in the study, 394 (2.4%) reported that at least one household member suffered from an unintentional injury in the past 12 months. The leading mechanisms of injury were unintentional falls (152 falls, 33.2%) and road traffic incidents (96 incidents, 21.0%). Among household members who were injured, 84.3% survived and 15.7% died because of the injury. Divorce marital status of the household head [AOR: 2.12, 95% CI (1.12–4.41)] and family size of the household ≥ 6 [AOR:1.65, 95% CI (1.21–2.26)] were associated with high likelihood of occurrence of an injury, while lowest household wealth index [AOR: 0.69, 95% CI (0.50–0.95)] was protective against injuries.

**Conclusion:**

A low prevalence of unintentional injury was found from the community in this survey, which might be due to the tendency of the community to report severe injuries. Fall and road traffic accidents were the leading mechanisms of selected sociodemographic factors of the households that were associated with unintentional injuries. Injury prevention efforts should focus on falls and transportation injuries with special attention to the sociodemographic context of the communities.

## 1. Introduction

More than 5 million people die each year as a result of injuries worldwide [1]. Injuries account for 9% of the world's deaths which is approximately 1.7 times the number of fatalities that result from HIV/AIDS, tuberculosis, and malaria combined [2]. Traffic collisions, falls, drowning, poisoning, and burns are commonly reported mechanisms of injury and mechanism that affect more than five million people globally every year [3]. Mortality rates due to unintentional injury are the highest in low-income countries [4].

Road traffic incidents and fall injuries are major public health issues in the world. A road traffic incident is the leading mechanism of injury accidents [5]. Approximately 1.35 million people die each year as a result of road traffic crashes [5]. Road traffic injuries are the main cause of death among people aged 5–29 years [5, 6]. Road traffic injury death rates are highest in the African region [7].

Fall injuries produce an estimated 646,000 fatalities each year, making them the second leading cause of death after road traffic incidents globally. Over 80% of fall-related fatalities occur in low- and middle-income countries. In all regions of the world, fall-related death rates are the highest among adults over the age of 60 years [8].

Other mechanisms of unintentional injuries including drowning, poisoning, and burns are becoming public health issues. Globally, over 320,000 died from drowning in 2016 [9]. Drowning is the third leading cause of death worldwide for children aged 5–14 years and over 90% of drowning deaths occur in low- and middle-income countries [9].

Injuries have been neglected from the health agenda for many years [10] and not improved despite being predictable and largely preventable [4]. The mechanism of injury varies from country to country and studies show the leading mechanism is also different for all age groups including young children [6, 11–17].

A community-based study from Bangladeshi, Saudi Arabia, Ghana, and Egypt revealed that the leading mechanism of injuries among children aged less than 18 years was an unintentional injury of falls followed by vehicle crashes, burns, and drown [11, 13, 18, 19]. Other population-based studies in Iran, Kenya, and Sudan among all age groups showed that unintentional injury from falls is the most common mechanism of injuries [20–22]. A similar study from Iran reported road incidents as a leading mechanism followed by bay fall [17], whereas in Kenya a study revealed cuts by objects are the most common injury type followed by falls and road incidents [20].

Global healthcare based studies from Bangladesh, Colombia, Malaysia, and Pakistan hospitals of children ages 0–12 years reported falls and road incidents as the most common mechanism of unintentional injury [6]. But also there are healthcare based studies in South Africa, Kenya, and Ethiopia that reported intentional injury including interpersonal violence and assault as leading mechanism followed by unintentional injury of road incidents [14–16, 23].

Ethiopia is a developing country and has no well-organized studies or an injury surveillance system [24]. The country's Strategic Plan on Violence and Injury Prevention and Emergency Medical Services Strategy has not emphasized injury prevention [25]. The data used in this strategy did not reflect the injury problem and its level to the far reach communities. Furthermore, many people in the community do not have access to medical services and have a low level of seeking medical care for the injuries and commonly manage injuries at the household level through traditional means. Hence, information about the burden of injury in the population is limited. Therefore, this study aimed to assess the unintentional injuries and their associated factors among households in Ethiopia.

## 2. Materials and Methods

This study was conducted in Ethiopia based on the 2016 Ethiopia Demographic and Health Survey (EDHS) data. The survey was regularly conducted every five years and collected health and health-related information at the household level from selected enumeration areas. The survey was administered by the Central Statistical Agency (CSA) in collaboration with the Federal Ministry of Health and collaborative partners [26].

The data collection tool used to gather information was a DHS Program's survey questionnaire and was adapted to reflect the population and health issues relevant to Ethiopia. The EDHS 2016 is the first time community-based information about injuries and incidents in Ethiopia. The EDHS 2016 collected information about the occurrence of injuries and incidents in a 12-month survey among 75,271 members who lived in 16,650 households [26].

Ethiopia is divided into nine geographical regions and two administrative cities [26, 27]. The sample was stratified and selected in two stages. Each region was also stratified into urban and rural areas. The sample size for the survey was designed to provide estimates of key health indicators for the country as a whole. The survey sample was representative at the national and regional levels and for urban and rural areas [26]. The sampling frame used for the 2016 EDHS is the Ethiopia Population and Housing Census (PHC), which was conducted in 2007 by the Ethiopia Central Statistical Agency [28]. The census frame is a complete list of 84,915 enumeration areas (EAs) created for the 2007 PHC. An EA is a geographic area covering an average of 181 households. The sampling frame contains information about the EA location, residence type (urban or rural), and the estimated number of residential households. Stratification and proportional allocation were achieved at each of the lower administrative levels by sorting the sampling frame within each sampling stratum before sample selection according to administrative units at different levels [26].

Initially, 645 enumeration areas (EAs) (202 from urban areas and 443 from rural areas) have been drawn using a probability proportional to size (PPS) from a complete list of 84,915 EAs. In the second stage of selection, a fixed number of 28 households per cluster were selected using a systematic random sampling approach. In the selected households, enumeration of all household members was made and information about the occurrence of injuries among all household members was collected primarily from the head of the household or his/her partner [26].

The EDHS data were collected from January 18, 2016, to June 27, 2016, using pretested questionnaires prepared in three major local languages (Amarigna, Tigrigna, and Oromiffa). The data collectors, editors, and supervisors received four weeks of training [26].

In the survey, respondents were asked whether any child or adult in the household was killed or injured in the past 12 months, and the injured person or caregiver could not carry out their normal activities for at least a day. Besides, information about the mechanism of injury, length of injury, severity (fatal or not), and characteristics of the victim (age, sex) was explored [26].

The “household” datasets of the DHS 2016 survey were downloaded from the DHS Program website in SPSS format [29]. The analyzed dataset is available from https://dhsprogram.com/Data/. The injury rates were presented as a percentage. Household Wealth Index was given scores based on the number and types of consumer goods they own, properties ranging from a television to a bicycle or car, source of drinking water, toilet facilities, and house flooring materials [29]. These scores were derived using principal component analysis. The association between selected sociodemographic characteristics and the unintentional injury was determined using multivariate analysis. Bivariate logistic regression and chi-square test were used to explore the presence of a statistical association between different independent variables and the outcome variable using crude odds ratio with 95% CI. A *p* value of <0.2 was used as a cutoff point to select the candidate variables for multivariable analysis. The cutoff point was selected to reduce unstable estimates in the multivariate logistic analyses [30, 31]. The collinearity test was checked by running collinearity diagnostics; collinearity of each variable in this study was less than 5. This indicates that a specified independent variable was not explained by another independent variable in the model [32]. Model fitting was checked using Hosmer–Lemeshow goodness of test which showed chi-squared test (*X*^2^ = 7.53) with a degree of freedom of 8 and a significance equal to 0.481. Finally, variables with a *p* value of less than 0.05 were considered statistically significant and presented by the adjusted odds ratio (AOR) with 95% CI in the multivariate analysis.

### 2.1. Operational Definition

#### 2.1.1. Unintentional Injury

Accidental injuries include road traffic, burns, drowning, poisonings, falls, and other injuries except deliberate injuries include violence/assault and self-inflicted Injuries [26, 33].

#### 2.1.2. Mechanism of Injury

Mechanism of injury refers to how the person was hurt including road traffic incidents, fire/burning, drowning, poisoning, kicked by cattle, and fall [26, 33].

#### 2.1.3. Household

Household consists of a person or group of persons, irrespective of whether related or not, who normally live together in the same household and housing units and have common cooking and eating arrangements [26, 27].

#### 2.1.4. Head of Household

Head of household is a person who provides economic support or manages the household and he/she is selected by household members for some reasons like his age or respect regardless of their sex [26, 27].

### 2.2. Ethical Considerations

The National Research Ethics Review Committee, Ministry of Science and Technology of Ethiopia, ethically cleared the EDHS 2016 study. Data were collected after obtaining informed consent from the respondents before participation in the study [26].

## 3. Results

### 3.1. Sociodemographic Characteristics Participant

The EDHS 2016 survey data were collected from 16,650 households across nine regions and two-city administration of Ethiopia. From the total of 18,008 sampled households, 17,067 had an occupant. Of the occupied households, 16,650 heads of household (respondents) were successfully interviewed, yielding a response rate of 98%. The majority (68.6%) of the households were from rural areas, while the remaining 31.4% were from urban areas.

The majority of respondents (68.5%) were men. The mean (±SD) age of the respondents was 44.2 (±16.2) years. About half (52.4%) of the respondents had no formal education and very few (9.5%) had attained a higher level of education. The median (interquartile range) of household size was 4 [3–6] (Table 1).

### 3.2. Injuries among Households

Heads of households reported at least one injury and the mechanisms of injury in the past 12 months; 394 (2.4%) and 64 (0.38%) respondents reported at least one household member affected with unintentional and intentional injuries, respectively.

Out of the total unintentional injured household members, 61.4% of victims were male. High injury incidents were reported from households with children aged 0–14 years accounting 105 (26.6%). Besides, unintentional injuries were high, 124 (31.5%) among households with the highest wealth index in quintile (Table 2).

### 3.3. Mechanism and Severity of Injuries

The leading mechanisms of injury were falls, 152 (38.6%), and road traffic incidents, 96 (24.4%). Among household members who experienced an unintentional injury in the past 12 months, 338 (85.8%) survived and 56 (14.2%) died. Concerning the length of lost working days, injured persons and the caregivers stay at home or health facility accounts 76 (22.8%) for less than 8 days, 115 (34.4%) between 8 and 30 days, and 96 (28.7%) between 1 and 6 months (Table 3).

Road traffic accidents were the leading mechanism of injury among age groups 15–24 and 25–34 years, while falls were a prominent mechanism of injury among age groups (0–14, 35–44, 45–54, 55–64, and >64). Drown and poisoning were the least mechanism of injury almost for all age groups (Table 4).

### 3.4. Sociodemographic Factors Associated with Injuries

Divorce marital status of household head [AOR: 2.12, 95% CI (1.02–4.41)] and family size of the household ≥6 [AOR:1.65, 95% CI (1.21–2.26)] were factors positively associated with injury and household members from the lowest household wealth index [AOR: 0.69, 95% CI (0.50–0.95)] were less vulnerable to unintentional injury (Table 5).

## 4. Discussion

This is the first population-based survey that provides national estimates on injuries in Ethiopia. A total of 394 (2.4%) respondents reported at least one household member was injured unintentionally from an incident. Head of household age, marital status of household head, presence of <5 years old children, family size, owning of land usable for agriculture, and owning livestock/farm animals were variables statistically significant with the unintentional injury.

In this study, the prevalence of unintentional injury for the last year was 2.4%. It is a lower incidence than studies in Sudan (8.2%) [21] and Iran (18.9%) [22]. This difference might be due to the inclusion of all individuals in both study and a person among households in the current study.

Falls and road traffic were the most common mechanism of unintentional injury, whereas poisoning and drowning were least the in the current study. It is consistent with a study among households of all age groups in Sudan [21] and children in low- and middle-income countries (6, 19). It might be due to the higher rates of physical activity while engaging in work such as cooking, cleaning, household maintenance, and caring for children without applying the safety practice [11, 22]. Also, fall injuries at home were more common among younger children due to a lack of adult supervision at home [6, 12, 13, 17, 19, 34]. Road traffic incident was the second leading mechanism for unintentional injury in the present study. It is consistent with other studies in Iran and Kenya [20, 22] and our findings provide additional views on the road as a dangerous place in Ethiopia [10, 16].

The current study additionally identified sociodemographic factors associated with unintentional injuries. The risk of an unintentional injury to household members from a divorced household head was two times more likely than single. The reason for this might be that divorced heads of households might suffer from varying psychological stressors [35]. These underlying conditions may compromise safety practices to prevent injury in households [36]. Households with more than five members have greater odds of injury when compared to households with less than five members. This finding is supported by other studies that family size is a predictor factor of injuries [37, 38]. It might be due to household crowding conditions [37]. The other explanation for such a finding might be because of a child's behavior, awareness of danger, child's parent injury counseling, and whether a child is left in the care of another child [36, 39].

The injury likelihood among members from the lowest household wealth index was lower than the highest tire. It is not consistent with studies that revealed low socioeconomic status is a risk factor for the occurrence of injury [14, 20, 21, 37]. The discrepancy of findings might be due to the difference in sociodemographic characteristics of study populations. Another explanation might be due to the fact that road traffic incident was the leading mechanism of unintentional injury among healthcare patients in Ethiopia [15] and Kenya [23]. Therefore, individuals from the highest household wealth index might have more exposure to road traffic because of mobile from place to place by vehicle. This might have a high chance of traffic-related injury than the lowest tire which have no access to transport due to lacking money or not owning car [40].

This study had its strengths and limitations. First, the study is population-based and used large data to assess unintentional injuries from valid nationally representative information. Such information has not been frequently presented in previous studies in Ethiopia. However, the DHS study data were from heads of households and only enumerates injuries that were severe enough to stop the normal activities that may exclude minor injuries. Secondly, the study only assessed the sociodemographic factors of injuries and did not explore more about household members' high-risk behaviors and other personal characteristics. Finally, we recommended further study, particularly using case-control design to explore more evidence on determinant factors associated with unintentional injury in the community.

In conclusion, a low prevalence of unintentional injury was found from the community in this survey and injuries among households are equally substantial as any public health problem in Ethiopia. Falls were the leading mechanism of injuries and road traffic incidents were second. Household head marital status, family size, and household wealth were variables statistically significant with an unintentional injury. Injury prevention efforts should focus on falls and transportation injuries with special attention to the sociodemographic context of the communities.

## Figures and Tables

**Table 1:** Sociodemographic characteristics of the respondents, Ethiopian Demographic and Health Survey, 2016.

Characteristics of respondents (*n* = 16,650)	Frequency	Percent
*Sex*		
Male	11413	68.5
Female	5237	31.5

*Age in years*		
15–24	1259	7.6
25–34	4192	25.2
35–44	3828	23.0
45–54	2725	16.4
55–64	2330	14.0
64+	2316	13.9

*Marital status of household head*		
Never married	1046	6.3
Married	12073	72.5
Widowed, divorced	3531	21.3

*Educational status*		
No education, preschool	8726	52.4
Primary	4658	28.0
Secondary	1686	10.1
Higher	1580	9.5

*Place of residence*		
Urban	5232	31.4
Rural	11418	68.6

*Number of household members*		
<4	6258	37.6
4–5	5024	30.2
≥6	5368	32.2

*Wealth index of household in quintile*		
Lowest	4676	28.1
Second	2348	14.1
Middle	2057	12.4
Fourth	2020	12.1
Highest	5549	33.3

**Table 2:** Proportion of unintentional injuries among affected household members, Ethiopian Demographic and Health Survey, 2016.

Injury status (*n* = 394)	Frequency	Percent
*Injuries by Sex*		
Male	242	61.4
Female	152	38.6

*Injuries by Age*		
0–14	105	26.6
15–24	58	14.7
25–34	71	18.0
35–44	50	12.7
45–54	40	10.2
55–64	28	7.1
64+	40	10.2
Unknown	2	0.5

*Injuries by residence*		
Urban	115	29.2
Rural	279	70.8

*Injuries by Region*		
Tigray	44	11.2
Afar	22	5.6
Amhara	58	14.7
Oromia	58	14.7
Somali	21	5.3
Benishangul	33	8.4
SNNPR	45	11.4
Gambela	36	9.1
Harari	13	3.3
Addis Adaba	35	8.9
Dire Dawa	29	7.4

*Injuries by household Wealth index in quintile*		
Lowest	96	24.4
Second	73	18.5
Middle	56	14.2
Fourth	45	11.4
Highest	124	31.5

**Table 3:** Mechanism and severity of unintentional injuries by sex, Ethiopian Demographic and Health Survey, 2016.

Mechanism and severity of injury	Frequency (%)		
	Male	Female	Total
*Mechanism of injury* (*n* = 394)			
Fall	99 (65.1)	53 (34.9)	152 (38.6)
Road incidents	58 (60.4)	38 (39.6)	96 (24.4)
Fire/Burn	25 (52.1)	23 (47.9)	48 (12.2)
Animal bite/Kicked	14 (56.0)	11 (44.0)	25 (6.3)
Drown and poisoning	5 (50.0)	5 (50.0)	10 (2.5)
Other	41 (65.1)	22 (34.9)	63 (16.0)

*Severity of injury* (*n* = 394)			
Injured and alive^#^	206 (61.7)	132 (39.5)	338 (85.8)
Dead due to injury	36 (64.3)	20 (35.7)	56 (14.2)

*Length of time unable to carry out normal daily activities* (*n* = 334)			
Less than 8 days	47 (61.8)	29 (30.2)	76 (22.8)
Between 8 to 30 days	78 (67.8)	37 (32.2)	115 (34.4)
Between 1 to 6 months	53 (55.2)	43 (44.8)	96 (28.7)
Longer than 6 months	25 (61.0)	16 (39.0)	41 (12.3)
Don't know	2 (33.3)	4 (66.7)	6 (1.8)

^#^ *One male and three female deaths were not due to injury and considered as alive after injury*.

**Table 4:** Mechanism of unintentional injuries by age group, Ethiopian Demographic and Health Survey, 2016.

Age		Mechanism of injury						^#^Total (*N* = 392)
		Road traffics incidents	Fire/Burn	Animal bite/Kicked	Fall	Drown and poisoning	Other	
0–14	Frequency (%)	11 (9.6)	27 (23.7)	6 (5.3)	48 (42.1)	2 (1.8)	11 (9.6)	105 (26.6)
15–24	Frequency (%)	26 (40.0)	6 (9.2)	0 (0.0)	18 (27.7)	2 (3.1)	6 (9.2)	58 (14.7)
25–34	Frequency (%)	24 (27.9)	6 (7.0)	3 (3.5)	14 (16.3)	2 (2.3)	22 (25.6)	71 (18.0)
35–44	Frequency (%)	12 (18.5)	4 (6.2)	4 (6.2)	20 (30.8)	0 (0.0)	10 (15.4)	50 (12.7)
45–54	Frequency (%)	10 (20.8)	2 (4.2)	3 (6.2)	19 (39.6)	1 (2.1)	5 (10.4)	28 (7.1)
55–64	Frequency (%)	5 (15.2)	1 (3.0)	4 (12.1)	15 (45.5)	0 (0.0)	3 (9.1)	40 (10.2)
>64	Frequency (%)	8 (20.0)	1 (2.5)	5 (12.5)	17 (42.5)	3 (7.5)	6 (15.0)	40 (26.6)

^#^ *Two persons' ages were not known and involved in the fall and drown mechanism of injury*.

**Table 5:** Association between sociodemographic factors and unintentional injury, Ethiopian demographic and health survey, 2016.

Variable name	Injury status (n = 16650)		COR (95% CI)	AOR (95% CI)
	Yes (%)	No (%)		
*Age of head of household*				
15–29	52 (1.5)	3310 (98.5)	1	1
30–64	291 (2.7)	10681 (97.3)	1.73 (1.29–2.34)^*∗*^	1.27 (0.91–1.77)
>64	51 (2.2)	2265 (97.8)	1.43 (0.97–2.12)	1.14 (0.74–1.75)

*Marital status of household head*				
Never married	10 (1.0)	1036 (99.0)	1	1
Married	297 (2.5)	11767 (97.5)	2.64 (1.4–4.97)^*∗*^	1.67 (0.84–3.31)
Widowed	52 (2.5)	2056 (97.5)	2.64 (1.34–5.22)^*∗*^	1.98 (0.96–4.10)
Divorced	35 (2.5)	2056 (97.5)	2.64 (1.30–5.35)^*∗*^	2.12 (1.02–4.41)^*∗*^

*Household has under* 5 *years old children*				
Yes	213 (2.6)	7853 (97.4)	1.26 (1.03–1.54)^*∗*^	1
No	181 (2.1)	8403 (97.9)	1	1.04 (0.82–1.33)
*Family size in household*				
<4	107 (1.7)	6151 (98.3)	1	1
4–5	121 (2.4)	4903 (97.6)	1.41 (1.09–1.85)^*∗*^	1.3 (0.98–1.75)
≥6	166 (3.1)	5202 (96.9)	1.83 (1.44–2.35)^*∗*^	1.65 (1.21–2.26)^*∗*^

*Owns land usable for agriculture*				
Yes	251 (2.7)	9196 (97.3)	1.34 (1.10–1.66)^*∗*^	0.82 (0.63–1.09)
No	143 (2.0)	7060 (98.0)	1	1

*Owns livestock, herds/farm animals*				
Yes	271 (2.6)	10307 (97.4)	1.27 (1.03–1.58)^*∗*^	0.92 (0.69–1.24)
No	123 (2.0)	5949 (98.0	1	1

*Wealth index in quintile*				
Lowest	96 (2.1)	4580 (97.9)	0.92 (0.70–1.20)	0.69 (0.50–0.95)^*∗*^
Second	73 (3.1)	2275 (96.9)	1.40 (1.05–1.88)^*∗*^	1.03 (0.71–1.48)
Middle	56 (2.7)	2001 (97.3)	1.22 (0.89–1.69)	0.88 (0.60–1.29)
Fourth	45 (2.2)	1975 (97.8)	1.00 (0.71–1.41)	0.73 (0.49–1.08)
Highest	124 (2.2)	5425 (97.8)	1	1

^*∗*^ *p* value less than 0.05.

## Data Availability

The datasets used and/or analyzed during the current study are available at https://dhsprogram.com/Data/.
